# Ecosystem Carbon and Nitrogen Accumulation after Grazing Exclusion in Semiarid Grassland

**DOI:** 10.1371/journal.pone.0055433

**Published:** 2013-01-30

**Authors:** Liping Qiu, Xiaorong Wei, Xingchang Zhang, Jimin Cheng

**Affiliations:** State Key Laboratory of Soil Erosion and Dryland Farming in the Loess Plateau, Northwest A&F University, Yangling, China; Jyväskylä University, Finland

## Abstract

The grazing exclusion in degraded grassland has been extensively used to prevent the loss of grassland resources and to improve grassland services. The effects of grazing exclusion on C and N balance, however, have not been well addressed but are essential for assessing grassland C sinks, the sustainable use of grassland resources and the support of grassland services. To understand the response of ecosystem C and N to grazing exclusion in semiarid grassland, we determined the C and N in litter, aboveground biomass, roots and soils from ungrazed grassland fenced at different times in northwest China. Our results showed that the aboveground biomass, root biomass and plant litter were 70–92%, 56–151% and 59–141% higher, respectively, in grazer excluded grassland than in grazed grassland. Grazing exclusion significantly increased C and N stored in plant biomass and litter and increased the concentrations and stocks of C and N in soils. Grazing exclusion thus significantly increased the C and N stored in grassland ecosystems. The increase in C and N stored in soil contributed to more than 95% and 97% of the increases in ecosystem C and N storage. The highest C and N stocks in ecosystems were observed in 17-year grazer excluded grassland. The results from this study indicate that grazing exclusion has the potential to increase C and N storage in degraded semiarid grassland and that the recovery of ecosystem C and N was mainly due to the accumulation of C and N in soils.

## Introduction

Grasslands cover 20% of the terrestrial surface and account for 20% or more of total terrestrial production [1]. Owing to their importance in socio-economics, culture, ecology and environmental quality, grassland ecosystems have become one of the most active subjects of research by ecologists around the world [2]. On a global scale, grasslands store more than 10% of terrestrial biomass C and 10–30% of global soil organic carbon (OC), and they have been estimated to sequester C in soil at a rate of 0.5 Pg C yr^−1^, accounting for about one-fourth of the potential carbon (C) sequestration in world soils [1], [3]. Carbon and nitrogen (N) are key factors determining the properties of ecosystems, such as primary productivity and plant diversity [4]–[6] and rates of decomposition [7], and therefore could be important mediators of the richness of functional groups and ecosystem functioning. Understanding the relationships between grassland management and the status of C and N is thus not only of academic interest but also crucial for the sustainable use of grassland resources and support of grassland services.

Previous studies in semiarid regions have shown that any changes in land use, such as introducing grazing and conversion to shrubland and agricultural land, will decrease soil and ecosystem C and N [8]–[10]. Possible mechanisms of the loss of C and N include changes in composition of plant communities, changes in plant roots and their control of the soil microbial community [11], [12], decreased input of organic material from aboveground biomass and roots due to biomass removal [13], decreased availability of soil resources in grasslands [14]–[17] and the disruption of soil structure resulting in losses of C and N through accelerated mineralization of soil organic matter and erosion by water and wind [9], [18]–[23].

The grazing exclusion of degraded grassland has been extensively employed in China and other regions to prevent its degradation and to improve grassland services [2], [24]. As an important conservation practice, grazing exclusion can have potential effects on the storage of C and N in grassland ecosystems due to the increased productivity of the biomass and the input of C and N to soils and ecosystems. The effects of grazing exclusion on C and N balance in grassland, however, have not been well addressed. We hypothesize that grazing exclusion in degraded grassland can increase C and N in soils and ecosystems because the removal of herbivores, particularly larger herbivores, can increase C and N stocks in ecosystems [25].

To test our hypothesis, we collected soil, plant, root and litter samples from fenced semiarid grasslands with fencing ages of 17-year, 22-year and 27-year (grazer excluded grassland) in a typical grassland of northwest China. We selected an adjacent unfenced grassland as a control (grazed grassland). The C and N concentrations in soils and plant samples were measured, and the stocks of C and N in different pooled samples and in the ecosystem were calculated to assess the response of C and N to grazing exclusion in semiarid grassland. The objective of this study was to understand how the C and N accumulate in semiarid grassland after grazing exclusion.

## Materials and Methods

### Ethics statement

The permit for soil and plant sampling was obtained from the Yunwushan Natural Grassland Management Bureau, which is responsible for the protection and management of the Yunwushan Natural Grassland Protection Zone.

### Study sites

This study was conducted in the Yunwushan Natural Grassland Protection Zone (36°13′–36°19′N; 106°24′–106°28′E) at Guyuan City, Ningxia Hui Autonomous Region, China. The grassland protection zone has an area of 6700 ha and an elevation ranging from 1800 to 2148 m. The study area has a continental monsoon climate. The mean temperature is 6.9°C. The maximum and minimum temperatures occur in July (24°C) and January (−14°C), respectively. The frost-free period is 124 d, normally beginning in mid-April and ending in late September. The mean annual precipitation is 425 mm. The soil in the study area is a montane grey-cinnamon soil classified as a Calci-Orthic Aridisol according to the Chinese taxonomic system, which is equivalent to a Haplic Calcisol in the FAO/UNESCO system.

### Field investigation and sampling

The study area was grazed by goats before protection. The intensity of grazing had varied over the years. In 1982, before protection, the physicochemical properties of the soils in the study area with the same soil type and similar natural conditions were not significantly different [26]. Three grazer excluded treatments established at different times were studied. Grassland sites were fenced with goat proof wire mesh year 1982, 1987 and 1992 and consequently goat grazing was excluded for 27 (GE27), 22 (GE22) and 17 years (GE17), respectively. The areas of the three treatments were 1200, 1000, and 1500 ha, respectively. A grazing area of 3000 ha was maintained as a control (CK). All four areas adjoined each other, and no fertilizer was applied. The dominant grass species in the grazer excluded and grazed grassland were bunge needlegrass (*Stipa bungeana*) and Russian wormwood (*Artemisia sacrorum Ledeb.*).

Most parts of the grazed grassland and grazer excluded grassland have similar topography and altitude. All the plots for both grazed and grazer excluded treatments have the same soil type and similar physiographic conditions (slope degree, slope direction, topography and altitude). Our previous study in the same control area showed that there were no changes in soil OC and N during past 27 years in the continuously grazed area (CK treatment) [9]. We therefore believe that the soils in each treatment had similar initial conditions and the existing grazed grassland can be used as the control to compare the effects of grazing exclusion on C and N accumulation in plants and soils.

**Figure 1:**
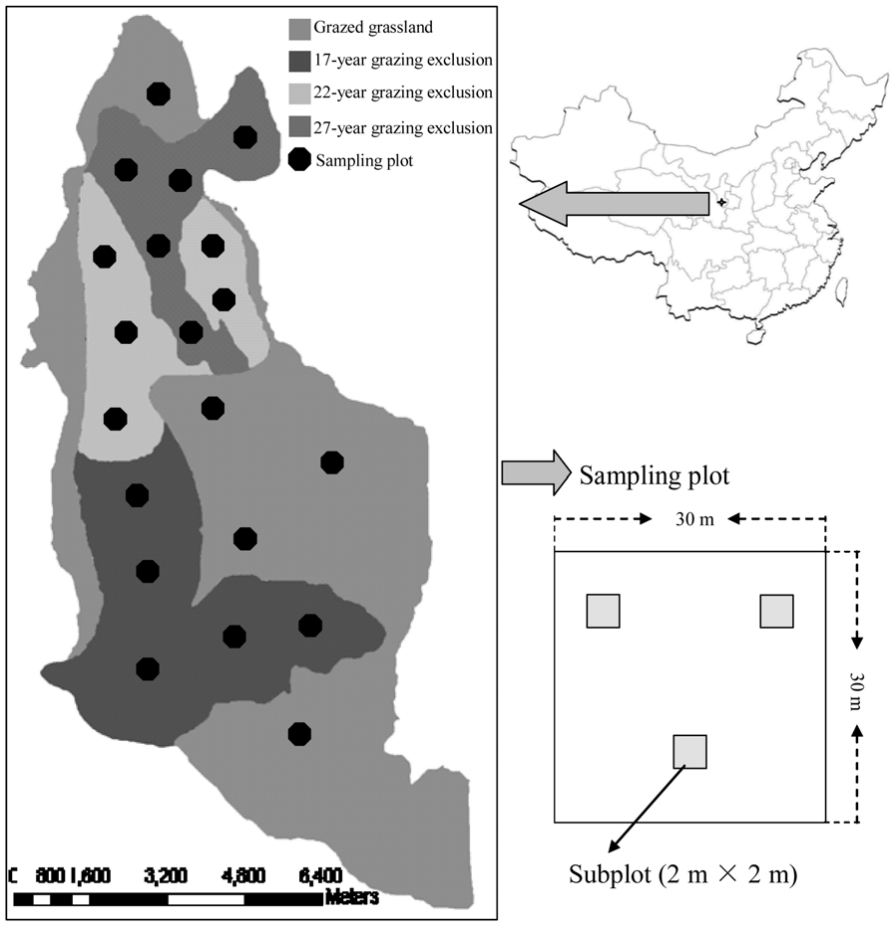
Location of the study site and sampling scheme.

Five pseudo-replicated plots (30 m×30 m) were randomly established within the grazed and grazer excluded treatments in August 2009 (Fig. 1). True replication was not possible in this study. The plots were at least 2 km apart. Considering that each treatment has the area larger than 1000 ha and the distance between each plot is larger than 2 km, our sampling design can reflect the effects of grazing exclusion on C and N in this ecosystem. Three 2 m×2 m subplots were established in each plot for field investigation and sampling. The subplots were at least 15 m apart and did not differ in physiographic conditions. In each subplot, the canopy cover and dominant species were determined. The canopy covers were 25%, 85%, 100% and 100% in grazed, 17-year grazing exclusion, 22-year grazing exclusion and 27-year grazing exclusion treatments, respectively. The aboveground biomass was harvested in August when the peak aboveground biomass of the two grass species occurred. The surface litter was also collected. Three profile samples were collected from each subplot for the measurement of root biomass. The samples (at depths of 0–10, 10–20, 20–40, 40–60, and 60–80 cm) were collected using a tube auger 9.0 cm in diameter. Roots were separated from the soil samples. The samples of litter, aboveground biomass and roots were washed with deionized water and dried at 65°C for 48 h. The dry samples were weighed and then ground to pass through a 0.25 mm sieve for measurement of C and N.

In each plot, a pit 1.0 m long×0.7 m wide×1.0 m deep was dug for measuring soil bulk density. The soil was sampled using a stainless-steel cutting cylinder (5.0 cm dia.×5.0 cm high) at depths of 0–10, 10–20, 20–40, 40–60 and 60–80 cm. The soil cores were dried at 105°C for 24 hours and then weighed for the calculation of soil bulk density. Three representative soil samples were randomly collected at the same depth increments as the bulk density samples in each subplot for the measurement of soil OC and N. All visible pieces of organic material were removed. The moist soil samples were brought to the laboratory, air-dried and ground to pass through a 0.25 mm sieve for the measurement of soil OC and N concentrations.

### Laboratory and data analyses

The C and N concentrations of litter, aboveground biomass, root samples, and OC and N concentrations in soils were analyzed using a VARIO EL III CHON analyzer (Elementar, Germany) at the Testing and Analysis Center of Northwest University, Xi'an, China.

The stocks of soil OC and N (Mg ha^−1^) were calculated as follows:

Stocks of OC*_i_* = D*_i_*×BD*_i_*×OC*_i_*/10 (1)

Stocks of N*_i_* = Di×BD*_i_*×N*_i_*/10 (2)

where D*_i_*, BD*_i_*, OC*_i_* and N*_i_* are the thickness (cm), bulk density (g cm^−3^), organic C (g kg^−1^) and N (g kg^−1^), respectively, of the *i*th layer of the soil.

A one-way analysis of variance followed by a Turkey's post hoc test was conducted using SAS version 8.0 to examine the effects of grazing exclusion on the C and N stored in litter, aboveground biomass, roots, and soils.

## Results

### 1. C and N in vegetation

**Figure 2:**
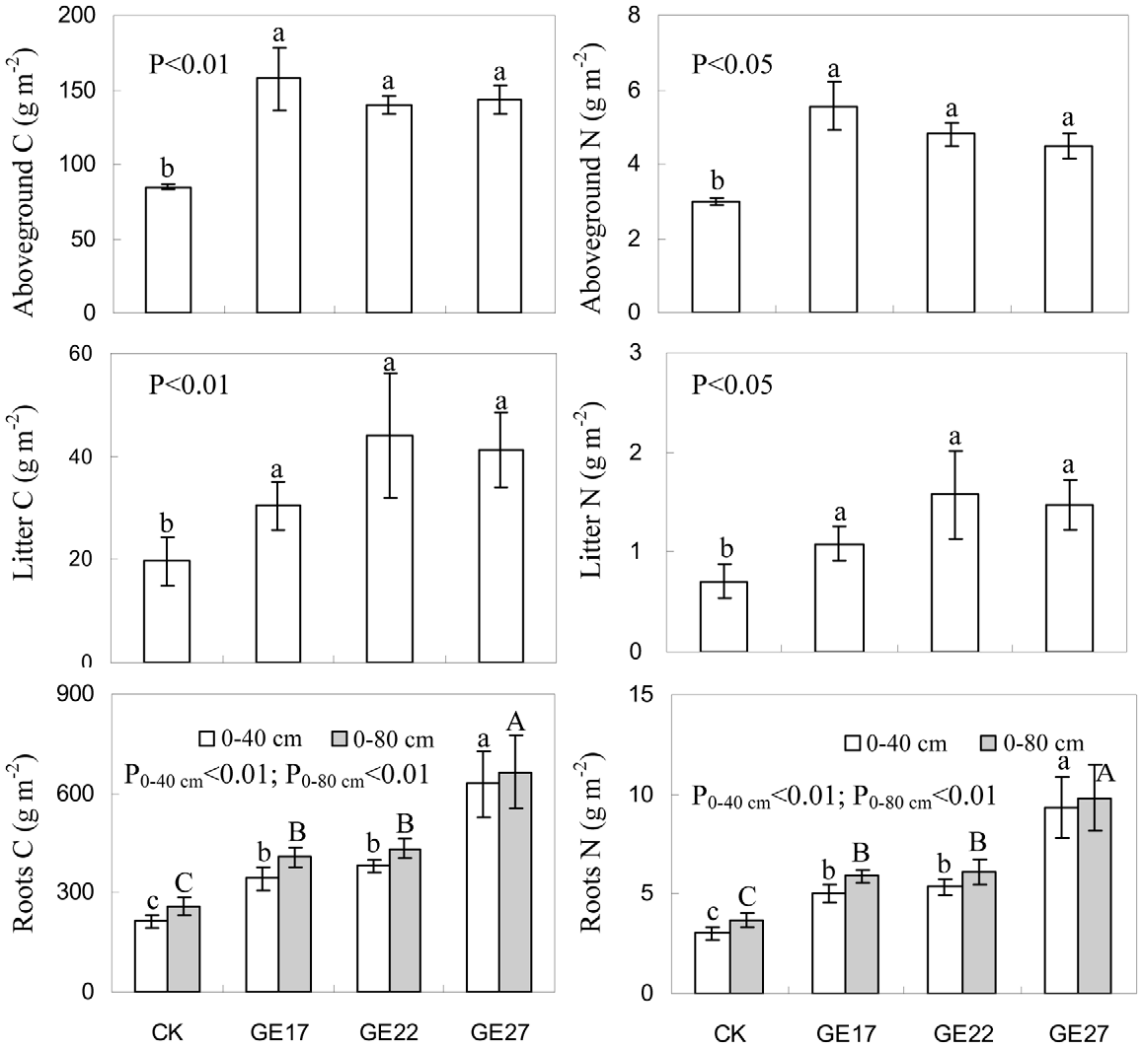
The effects of grazing exclusion on surface litter, aboveground biomass and roots biomass in grazed grassland. Error bars are the standard error of the mean. For aboveground biomass, surface litter and roots biomass in the 0–40 cm layer, means with different lowercase letters are different at P<0.05. For roots biomass in the 0–80 cm layer, means with different uppercase letters are different at P<0.05. (CK: grazed grassland; GE17: 17-year grazing exclusion; GE22: 22-year grazing exclusion; GE27: 27-year grazing exclusion).

Grazing exclusion significantly enhanced the accumulation of above- and belowground biomass and plant litter in the ecosystem (Fig. 2). The amount of aboveground biomass, belowground biomass and plant litter accumulated on the surface of the soils in the grazer excluded grassland ranged from 3.2 to 3.7, 10.2 to 16.3 and 0.7 to 1.0 Mg ha^−1^, respectively, which were 70–92% (P<0.05), 56–151% (P<0.05) and 59–141% higher (P<0.05), respectively, than those in the grazed grassland. The root biomass was mainly distributed in the 0–40 cm soil layer, accounting for 75–96% of the root biomass in the top 80 cm of soil. The root biomass in the 0–40 cm soil layer was 53–183% higher in the grazer excluded grassland than in the grazed grassland (P<0.05).

**Figure 3:**
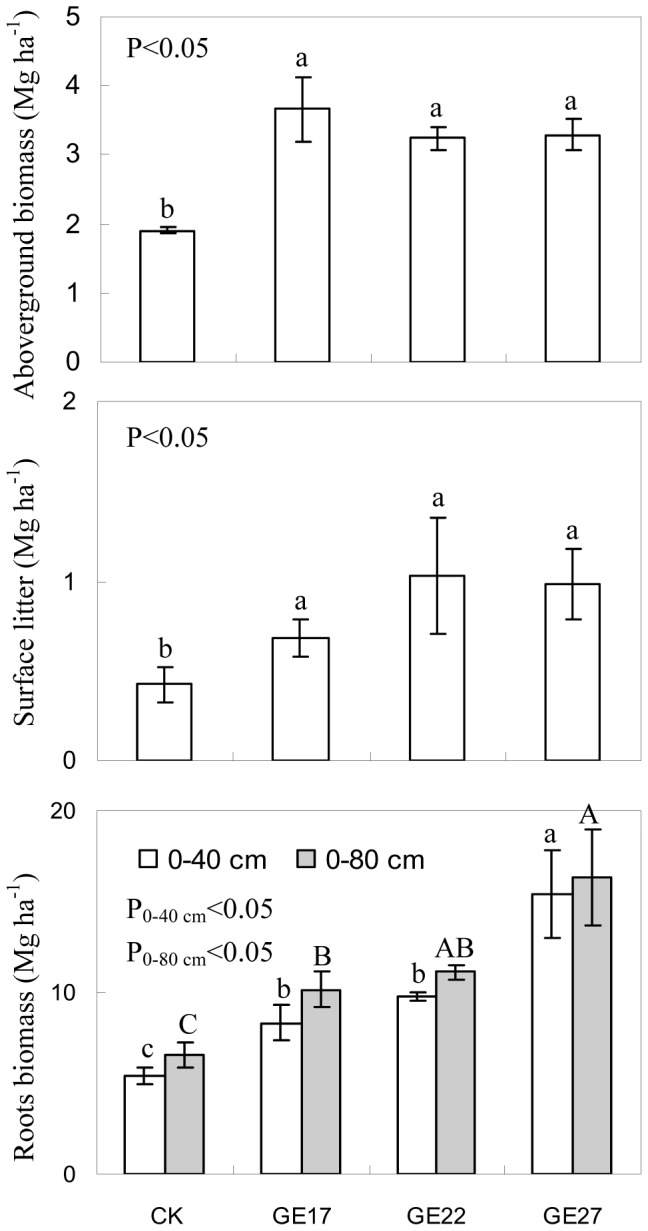
The effects of grazing exclusion on C and N stored in litter, aboveground biomass and roots in grazed grassland. Error bars are the standard error of the mean. For C and N stored in aboveground biomass, surface litter and roots biomass in the 0–40 cm layer, means with different lowercase letters are different at P<0.05. For C and N stored in roots biomass in the 0–80 cm layer, means with different uppercase letters are different at P<0.05. (CK: grazed grassland; GE17: 17-year grazing exclusion; GE22: 22-year grazing exclusion; GE27: 27-year grazing exclusion).

Grazing exclusion significantly increased C and N stored in plant biomass and litter (Fig. 3). The C stocks in aboveground biomass, belowground biomass and litter were 64–86% (P<0.01), 58–157% (P<0.01) and 55–125% higher (P<0.01), respectively, in grazer excluded grassland than in grazed grassland, while the N stocks were 51–87% (P<0.05), 58–157% (P<0.05) and 55–125% higher (P<0.05), respectively. Similar to root biomass, the C and N stored in roots were mainly in the 0–40 cm layer, accounting for 83–95% of the root C and N in the top 80 cm of soil, and were 61–194% higher in grazer excluded grassland than in grazed grassland (P<0.01). The C and N stocks in the aboveground biomass were not affected by grazing exclusion age, but the C and N stocks in the root biomass increased with grazing exclusion age. These results suggest that grazing exclusion has the potential to accumulate C and N in grass biomass and litter in semiarid grassland.

### 2. C and N in soils

**Figure 4:**
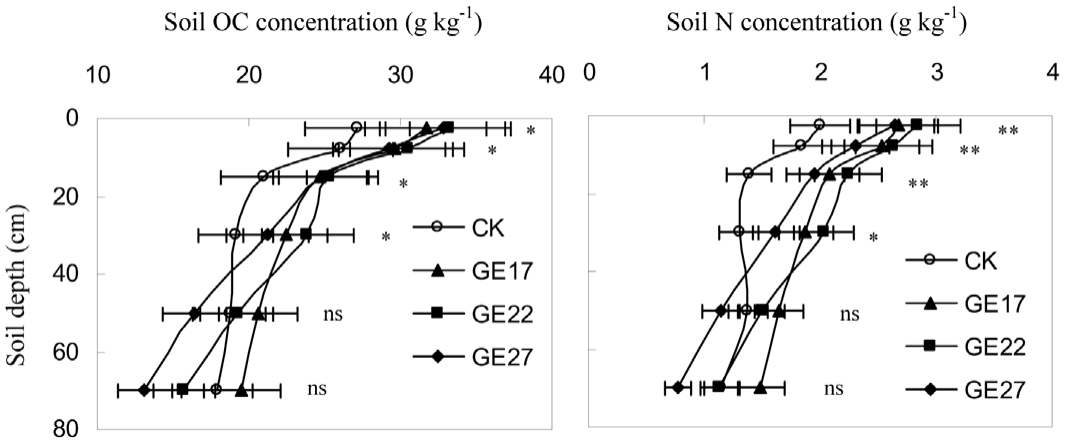
The effects of grazing exclusion on OC and N concentrations in soils of grazed grassland. Error bars are the standard error of the mean. (*: P<0.05; **: P<0.01; ns: P>0.05; CK: grazed grassland; GE17: 17-year grazing exclusion; GE22: 22-year grazing exclusion; GE27: 27-year grazing exclusion).

**Figure 5:**
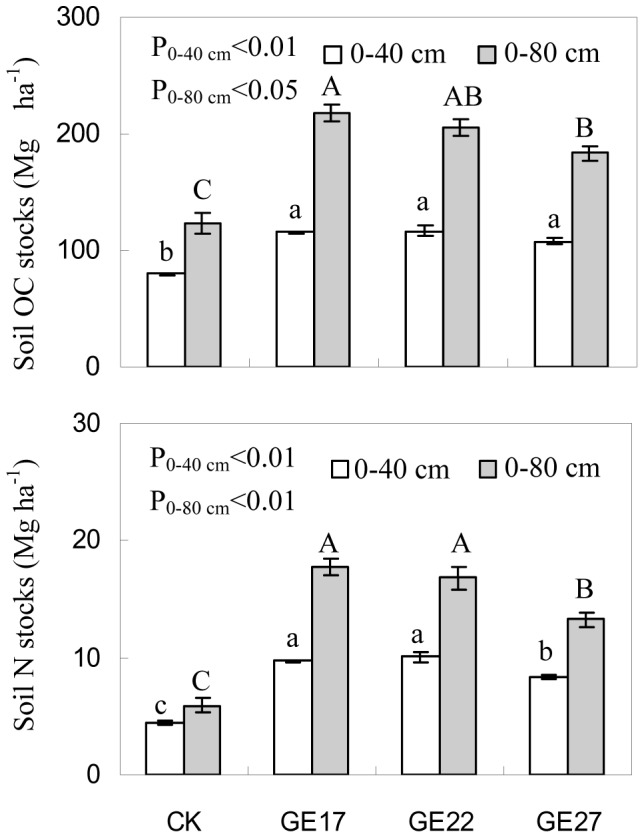
The effects of grazing exclusion on OC and N stocks in soils of grazed grassland. Error bars are the standard error of the mean. For soil OC and N stocks in 0–40 cm and 0–80 cm soil layers, means with different lowercase and uppercase letters are different at P<0.05, respectively. (CK: grazed grassland; GE17: 17-year grazing exclusion; GE22: 22-year grazing exclusion; GE27: 27-year grazing exclusion).

Grazing exclusion significantly increased OC and N concentrations (Fig. 4) and stocks (Fig. 5) in the 0–80 cm soil layer. The OC and N concentrations were 6–14% (P<0.01) and 15–37% higher (P<0.01), respectively, in grazer excluded grassland than in grazed grassland, and the OC and N stocks were 49–77% (P<0.05) and 125–199% higher (P<0.05), respectively, in grazer excluded grassland than in grazed grassland. Additionally, the OC and N stored in the 0–40 cm layer accounted for more than half of the OC and N stored in the top 0–80 cm of soil (53–65% for OC and 55–75% for N). These results suggest that grazing exclusion has the potential to sequester C and N in soils and that the potential was larger in the 0–40 cm soil layer than in deeper soils. The effects of grazing exclusion on soil C and N concentrations and stocks, however, varied with grazing exclusion age, with the highest C and N in 17-year grazing exclusion treatment, indicating a peak accumulation and maximum sequestration of C and N in soils around 17 years after grazing exclusion.

### 3. C and N in the ecosystem

**Figure 6:**
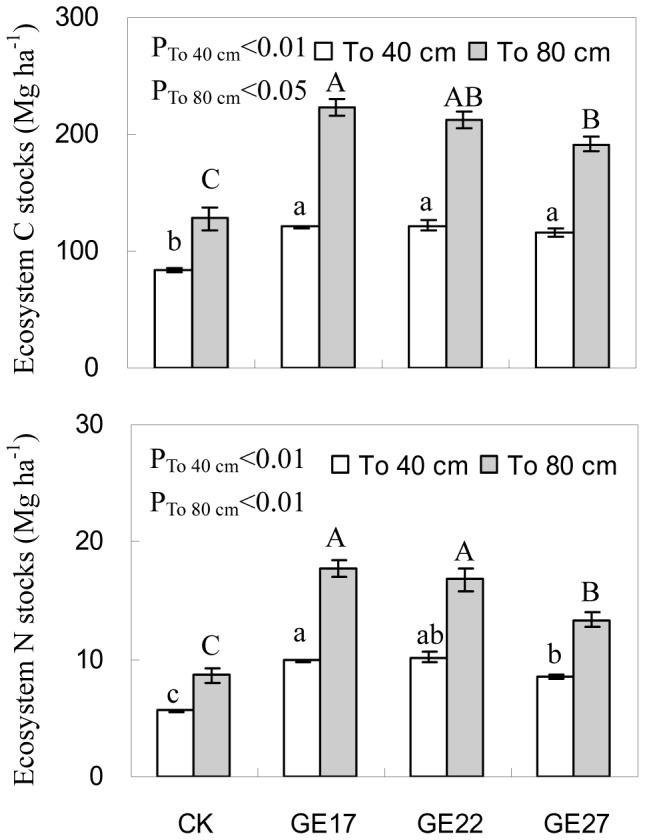
The effects of grazing exclusion on C and N in ecosystems (aboveground+belowground) in grazed grassland. Error bars are the standard error of the mean. For ecosystem C and N stocks down to 40 cm and 80 cm depths, means with different lowercase and uppercase letters are different at P<0.05, respectively. (CK: grazed grassland; GE17: 17-year grazing exclusion; GE22: 22-year grazing exclusion; GE27: 27-year grazing exclusion).

Grazing exclusion significantly increased the storages of C and N in the ecosystem, which were 50–75% (P<0.01) and 55–106% higher (P<0.01), respectively, in grazer excluded grassland compared to grazing grassland (Fig. 6). For both grazer excluded and grazed systems, more than 95% of C and 97% of N were stored in the soil, while less than 1% of C and N were stored in aboveground biomass and litter. Additionally, the highest C and N stocks in the ecosystem were observed in 17-year grazing exclusion treatment, suggesting a peak accumulation of C and N in semiarid grassland around 17 years after grazing exclusion.

## Discussion

Our results demonstrate a significant increase of C and N in soils and ecosystems after grazing exclusion in grassland, supporting our hypothesis that grazing exclusion has the potential to increase C and N storage in degraded semiarid grassland. Grazing exclusion resulted in an accumulation of 2.4–5.6 Mg C ha^−1^ yr^−1^, greater than the estimated global sequestration rate of C for grassland and semiarid grassland, and an accumulation of 0.18–0.54 Mg N ha^−1^ yr^−1^. The estimated global sequestration rate of C of grassland ranges from 0.003 to 0.057 Mg ha^−1^ yr^−1^ [27], [28]. These estimates do not consider the effects of excluding grazing. Our higher values for the accumulation of C suggest that grazing exclusion may accelerate the sequestration of C, at least in semiarid regions. The accumulation of C and N in grassland ecosystems can be directly attributed to the increase of above- and belowground biomass, the accumulation of grass materials as litter on the ground and the increased input of C and N into soils by litter and roots. This accumulation can also be indirectly attributed to the decreased loss of biomass C and N from the exclusion of disturbance, and the decreased loss of soil C and N by reducing soil respiration, and ammonium volatilization and N_2_O emission resulting from nitrification [29]–[34].

The effect of grazing on changes in C and N in grassland has received much attention, but the accumulation of C and N in the ecosystem after grazing exclusion in grassland has rarely been reported. Grazing significantly reduces C and N stored in grassland ecosystems. Bagchi and Ritchie (2010) reported a 32–33% decrease in aboveground C input and a 21–63% decrease in belowground C input to ecosystems with grazing [12]. Schuman et al. (1999) observed 20–52% and 7–16% decreases of C and 15–30% and 18–52% decreases of N in aboveground biomass and roots (0–60 cm depth), respectively, after 12 years of grazing on a native mixed grassland in Wyoming [35]. In our grazer excluded grassland, the aboveground C and N input (including litter) to the ecosystem increased by 76–80% and 62–81%, respectively, while the belowground C and N input to the ecosystem increased by 50–75% and 55–106%, respectively. The relatively larger increase in C and N by grazing exclusion compared to the decrease in C and N by grazing suggest that the recovery of C and N in degraded grassland could be faster than expected.

Grazing exclusion significantly increased both concentrations and stocks of C and N in the soils of grassland. Concentrations increased by 0.05–0.18 g C kg^−1^ yr^−1^ and 0.01–0.03 g N kg^−1^ yr^−1^, and stocks increased by 0.21–0.55 Mg C ha^−1^ 10 cm^−1^ yr^−1^ and 0.02–0.05 g N ha^−1^ 10 cm^−1^ yr^−1^. These rates were relatively larger than the losses of soil C and N due to grassland grazing or conversion to other types of land use. For example, Wang et al. (2012) reported a 20–37% decrease in soil OC and a 5–37% decrease in soil N after light and heavy grazing on the northern Tibetan Plateau, corresponding to losses of 0.14–0.29 Mg C ha^−1^ 10 cm^−1^ yr^−1^ and 0.004–0.013 Mg N ha^−1^ 10 cm^−1^ yr^−1^ [36]. Ingram et al. (2008) reported that 21 years of heavy grazing resulted in losses of 0.10 Mg C ha^−1^ 10 cm^−1^ yr^−1^ and 0.009 Mg N ha^−1^ 10 cm^−1^ yr^−1^ in the 0–60 cm soil layer in a mixed-grass ecosystem [37]. Qiu et al. (2012) reported a 58% decrease in soil OC after conversion of grassland to cropland in the Loess Plateau, representing a loss of 0.30 Mg C ha^−1^ 10 cm^−1^ yr^−1^ [9]. The accumulation of soil OC and N after grazing exclusion in grassland should thus be greater than the loss of OC and N by grazing or conversion to agriculture, and rehabilitation of degraded grassland after stopping any disturbance should be rapid.

The accumulation of C and N in soils after grazing exclusion can be explained by increased input and decreased loss of C and N. We observed significant positive relationships between soil C and N concentrations and root biomass (*r* = 0.653, *P*<0.001 for C; *r* = 0.669, *P*<0.001 for N; n = 100), in agreement with the finding that grazing triggers the loss of soil C by altering plant roots and their control on soil microbes [38] and thus in support of our explanation that grazing exclusion enhances the input of soil C and N. Additionally, many studies have shown that disturbance of grassland increases the loss of C and N through respiration and N_2_O emission and that these losses are reduced when the disturbance is stopped. For example, grazing can significantly increase soil C mineralization, nitrification and ammonification [29], enhancing soil respiration rates [30], ecosystem respiration rates [31], [32] and N_2_O emission [33], [34], which result in the loss of soil OC and N. Additionally, the effects of grazing on mineralization of soil OC and N were seasonally dependent and were characterized by increased mineralization during the growing season and decreased mineralization during the non-growing season [39], [40]. These findings indirectly support our explanation that grazing exclusion reduces losses of soil OC and N. The increase in OC and N stored in the soil contributed to 92–98% and 97–99% of the increases in ecosystem C and N storage, respectively, suggesting that the recovery of ecosystem C and N mainly occurred by the accumulation of C and N in soils after grazing exclusion in grassland.

The effects of grazing exclusion age on C and N stored in both soils and ecosystems indicated a maximum for C and N accumulation. Such a maximum amount of C and N accumulation represents the potential of C and N sequestration of degraded grassland at the study site. The existence of a maximum accumulation of C and N in soils and ecosystems also suggests that after certain periods of grazing exclusion (17-year in this study), the soils and ecosystems will no longer accumulate C and N, indicating that the grassland in the study area was stable or mature, in terms of their C and N storage, after 17-year of grazing exclusion. Other studies have found that grasslands in northern China subjected to 20-year grazing exclusion were stable in their productivity [41], [42], soil respiration [43] and C and N storage [22], [44]. The decrease of C and N after 17-year grazing exclusion may be due to increased productivity driving greater competition for resources such as nutrients, water, light and heat. Low availability of such resources can feedback to the increased microbial demand of resources, which in turn enhances the mineralization of soil organic material and thus the loss of C and N [22], [45]. An increased accumulation of fresh litter and organic material may also prevent the infiltration of rainfall into the soil. The interception of rainfall by accumulated litter and organic material accelerates their decomposition and renders the rainfall unavailable for infiltration, plant growth and cycling of C and N in semiarid and arid regions [22], [46], [47]. This interception of rainfall results in increased losses of biomass C and N and a decreased return of C and N through litter and organic material to the soil. Grassland in arid and semiarid regions could thus be properly used after accumulating maximum C and N so as to maintain the function of C and N sequestration. Our results demonstrated the accumulation of C and N in semiarid grassland after relatively long-term (17 to 27 years) grazing exclusion, however, short-term effects of grazing exclusion on C and N in grassland should be further examined so as to establish C and N dynamics following grazing exclusion in semiarid grazed grassland.
