# Correction: Ecosystem Carbon and Nitrogen Accumulation after Grazing Exclusion in Semiarid Grassland

**DOI:** 10.1371/annotation/7f3bd42b-6c85-4d65-a117-c90b18d39154

**Published:** 2013-06-21

**Authors:** Liping Qiu, Xiaorong Wei, Xingchang Zhang, Jimin Cheng

The legends for Figures 2 and 3 were incorrectly switched. The legend that appears with Figure 2 should be with Figure 3 and the legend that appears with Figure 3 should be with Figure 2. The figures themselves are in correct order.

